# Diagnostic accuracy of a deep learning model for pterygium detection in Barcelos, Brazilian Amazon

**DOI:** 10.5935/0004-2749.2025-0053

**Published:** 2025-09-10

**Authors:** Diego Casagrande, Mauro Gobira, Arthur G. Fernandes, Marcos Jacob Cohen, Paula Marques Marinho, Kevin Waquim Pessoa Carvalho, Ariane Luttecke-Anders, Beatriz Araujo Stauber, Nívea Nunes Ferraz, Jacob Moysés Cohen, Adriana Berezovsky, Solange Rios Salomão, Rubens Belfort Jr.

**Affiliations:** 1 Instituto da Visão, Instituto Paulista de Estudos e Pesquisas em Oftalmologia, São Paulo, SP, Brazil; 2 Department of Ophthalmology and Visual Sciences, Escola Paulista de Medicina, Universidade Federal de São Paulo, São Paulo, SP, Brazil; 3 Division of Ophthalmology, Department of Surgery, Faculdade de Medicina, Universidade Federal do Amazonas, Manaus, AM, Brazil; 4 Pontificia Universidad Católica de Valparaíso, Valparaíso, Chile

**Keywords:** Pterygium/diagnostic imaging, Smartphone, Diag nostic techniques, ophthalmological, Deep learning, Tele me dicine, Artificial intelligence, Cross-sectional studies, Brazil/epidemiology

## Abstract

**Purpose:**

This pilot study evaluated the diagnostic accuracy of a deep learning model for detecting pterygium in anterior segment photographs taken using smartphones in the Brazilian Amazon. The model’s performance was benchmarked against assessments made by experienced ophthalmologists, considered the clinical gold standard.

**Methods:**

In this cross-sectional study, 38 participants (76 eyes) from Barcelos, Brazil, were enrolled. Trained nonmedical health workers captured high-resolution anterior segment images using smartphones. These images were analyzed using a deep learning model based on the MobileNet-V2 convolutional neural network. Diagnostic metrics–including sensitivity, specificity, accuracy, positive predictive value, negative predictive value, and area under the receiver operating characteristic curve–were calculated and compared with the ophthalmologists’ evaluations.

**Results:**

The deep learning model achieved a sensitivity of 91.43%, specificity of 90.24%, positive predictive value of 88.46%, negative predictive value of 92.79%, and an area under the curve of 0.91. Logistic regression revealed no statistically significant association between pterygium and demographic variables such as age or gender.

**Conclusions:**

The deep learning model demonstrated high diagnostic performance in identifying pterygium in a remote Amazonian population. These preliminary findings support the potential use of artificial intelligence–based tools to facilitate early detection and screening in underserved regions, thereby enhancing access to ophthalmic care.

## INTRODUCTION

Pterygium is a common fibrovascular growth of the conjunctiva that can progressively encroach onto the cornea and cause significant visual impairment if left untreated^(1^,^2)^. Its etiology is multifactorial, involving genetic susceptibility, environmental exposure, and other contributory factors, with ultraviolet (UV) radiation identified as a key driver of disease development and progression^(1^-^4)^.

According to a 2018 systematic review and meta-analysis, the global prevalence of pterygium is approximately 12%^(5)^. However, this prevalence varies widely by geographic region. For instance, high rates have been reported in the Brazilian Amazon (approximately 59%) and parts of China (39%), while substantially lower rates are observed in countries such as Australia (2.83%) and Iran (1.3%)^(6^-^9)^. The condition is particularly common in equatorial regions, where UV radiation exposure is intense^(1^-^4)^.

The Brazilian Amazon, characterized by high ambient UV levels and limited access to eye care services, demonstrates an especially high burden of pterygium. The Brazilian Amazon Region Eye Survey, conducted by our research group, remains the largest population-based study to report the highest known prevalence of pterygium to date^(6)^. In this cross-sectional study, carried out in Parintins (Amazonas State), pterygium emerged as the second leading cause of visual impairment and blindness, following uncorrected refractive error^(6)^.

In recent years, artificial intelligence (AI), particularly deep learning (DL), has gained traction in the medical field as a valuable tool for diagnosis and disease management^(10)^. AI applications in ophthalmology have enabled automated detection, classification, and monitoring of ocular diseases, including pterygium, especially in settings where access to slit-lamp biomicroscopy and trained specialists is scarce^(11^,^12)^. These technologies, when integrated with accessible tools such as smartphones, offer promising solutions for improving early diagnosis in remote and underserved areas^(6)^.

Nevertheless, challenges persist. Variability in image quality, limited availability of large and diverse datasets, and inconsistent validation approaches have hindered the diagnostic performance of some AI models^(13)^. In response, researchers have developed models trained on various imaging modalities–including slit-lamp and smartphone-captured images–and hybrid models that combine both. Many of these have demonstrated promising diagnostic performance, often comparable with that of experienced ophthalmologists^(11^,^12^,^14^,^15)^.

Given the disproportionately high prevalence of pterygium in the Brazilian Amazon and the limited access to ophthalmic care in this region, there is a compelling need for scalable and accurate screening tools. This pilot study was conducted to evaluate the feasibility and initial diagnostic performance of a DL model for pterygium detection. Specifically, we assessed the model’s performance using smartphone-acquired anterior segment photographs, comparing its diagnostic accuracy with that of trained health workers and ophthalmologists–the clinical gold standard–in a population from the Brazilian Amazon.

## METHODS

### Ethical approval

This study was conducted in accordance with the principles of the Declaration of Helsinki and was appro ved by the appropriate institutional review board (details omitted for double-anonymized peer review). Informed consent was obtained from all participants prior to data collection.

### Study design

This pilot study employed a cross-sectional design to assess the diagnostic accuracy of a DL model for detecting pterygium using smartphone-acquired anterior segment images. The model’s performance was compared against the diagnostic consensus of experienced ophthalmologists, considered the clinical gold standard.

Participants were recruited from the population of Barcelos, a semirural municipality in the state of Amazonas, Brazil. Eligible participants included individuals aged 18 yr or older who sought care at one of three strategically selected basic health units (BHUs), each with an average daily patient volume of approximately 50. All individuals presenting through spontaneous demand were considered for inclusion. Exclusion criteria included patients without ocular complaints, whose smartphone-acquired images were deemed normal by both the nonmedical health worker and the attending ophthalmologist.

### Data collection

Prior to initiating the study, a specialized training session was conducted for four nonmedical health workers (nursing technicians) employed at the participating BHUs. The session was led by a highly qualified ophthalmic technologist (A.G.F.), who holds a Ph.D. in Visual Sciences with a specialization in pterygium. The training was divided into two distinct phases.

The first phase comprised didactic lectures delive red in person using PowerPoint presentations. These lectures were developed and adapted by researchers from the Graduate Program in Visual Sciences at the *Universidade Federal de São Paulo* (UNIFESP), *Escola Paulista de Medicina* (N.N.F. and A.B.). The key topics covered included the following:

– Basic anatomy and physiology of vision: Overview of the primary ocular structures and their respective functions.
– Common ocular complaints: Discussion of prevalent symptoms such as unilateral or bilateral vision loss, red eye, fleshy conjunctival growths, and leukocoria.
– External eye examination: Instruction on inspecting the external eye, with emphasis on identifying abnormalities such as pterygium and cataracts.
– Referral protocols: Guidelines for referring patients for further evaluation based on initial findings.

The second phase focused on practical training in smartphone-based anterior segment imaging. Participants were trained to capture high-quality ocular images and formulate preliminary diagnostic hypotheses for anterior segment conditions, specifically pterygium and cataracts. Each technician was assigned a unique identifier for tracking purposes. For each eye, a standardized examination form was completed, and a preliminary diagnosis was recorded.

These forms, along with the corresponding images, were subsequently reviewed by a team ophthalmologist stationed at the BHUs. Importantly, this ophthalmologist had no prior interaction with the technicians to ensure objective assessment. The ophthalmologist confirmed or revised the technicians’ diagnostic impressions. All study participants were then referred for comprehensive ophthalmological evaluation at the General Hospital of Barcelos (Nazaré Lacerda Chaves).

Smartphone-based imaging training included the use of two Samsung Galaxy A55 smartphones, selected for their high-resolution cameras, affordability, and availability. The training lasted approximately 2.5 h and was again led by the ophthalmic technologist (A.G.F.). It emphasized standardization to ensure image consistency across patients. Technicians were instructed to use the phone’s autofocus and to center the nasal and temporal palpebral fissures clearly within the frame. The focal point was to be properly aligned on the device screen for all cases to ensure uniformity.

**Figure 1:**
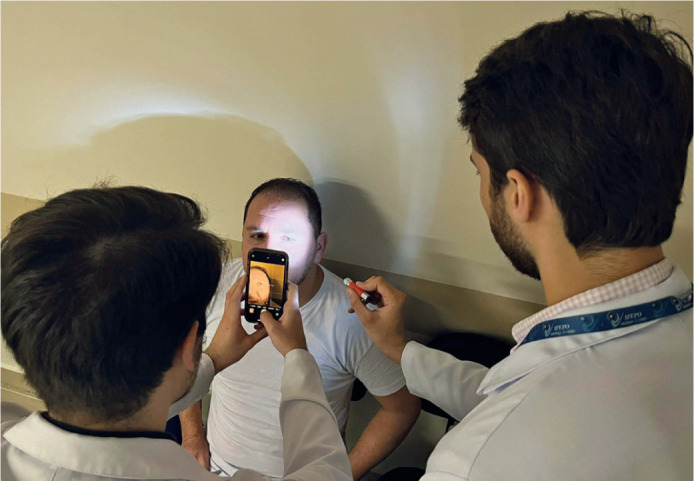
A technician captures an ocular image using a smartphone while a flashlight, angled at 45° to the temporo-nasal axis, enhances illumination of the pupil, providing a clearer view of the ocular surface.

After 1 day of practice, limitations in image quality were identified, prompting a protocol adjustment. From the second day onward, an auxiliary light source–a flashlight–was introduced. Positioned at a 45° to the temporo-nasal axis and directed toward the pupil, this light enhanced ocular surface illumination, resulting in clearer, more defined images. All subsequent photographs adhered to this revised protocol, incorporating both the flashlight enhancement and standardized alignment (Figure 1).

The comprehensive ophthalmological evaluation at the General Hospital of Barcelos included the following procedures:

– Distance visual acuity (DVA): Measured using an ETDRS logMAR chart at a 4-m distance, with participants wearing their habitual correction, if available. The smallest line on which at least four of five optotypes were correctly identified was recorded monocularly. Presenting DVA worse than 0.05 (<20/400) was classified as distance visual impairment.
– Near visual acuity: Assessed with the ETDRS “E” logMAR near vision chart under ambient lighting. Participants with bifocals or reading glasses were tested using their correction; others were asked to remove distance-only glasses. Monocular visual acuity worse than 0.05 (<20/400) was categorized as near visual impairment.
– Refraction: Automated refraction was performed using the Gilras GRK 7000 autorefractor. Best-correc ted visual acuity for both distance and near vision was subsequently determined.
– Anterior segment examination: Conducted using a Luxvision SL 1000 slit-lamp, this exam evaluated the eyelids, globe, pupillary reflexes, and lens. Pterygium cases were noted based on lesion size and location, while cataracts were graded using the Lens Opacities Classification System III.
– Posterior segment examination: Fundoscopy under pharmacologic pupil dilation was performed, employing both direct and indirect methods, to assess the macula and optic disc for pathological changes.

All patient data were anonymized, and personal identifiers were replaced with unique alphanumeric codes to maintain confidentiality. This anonymization process was strictly adhered to throughout the study, ensuring ethical compliance and the protection of participant privacy.

### Dataset preparation

In August 2024, horizontal cross-sectional anterior segment images were collected from patients diagnosed with pterygium at BHUs. Two experienced ophthalmologists (M.J.C. and D.C.) independently reviewed and classified these images into two categories based on the presence or absence of pterygium. Ultimately, a dataset comprising 170 anterior segment images from 85 patients was compiled and saved in JPEG format for training the DL model.

To ensure compatibility with standard DL model input requirements, all images were resized to 224 × 224 pixels and centered on the pupil. Patient-identifiable information and device metadata were removed to maintain confidentiality and avoid bias. The training and testing datasets were completely independent. The testing dataset included 124 anterior segment images from 62 patients (124 eyes), ensuring no overlap with training data.

### Gobvision AI platform and model training

**Figure 2:**
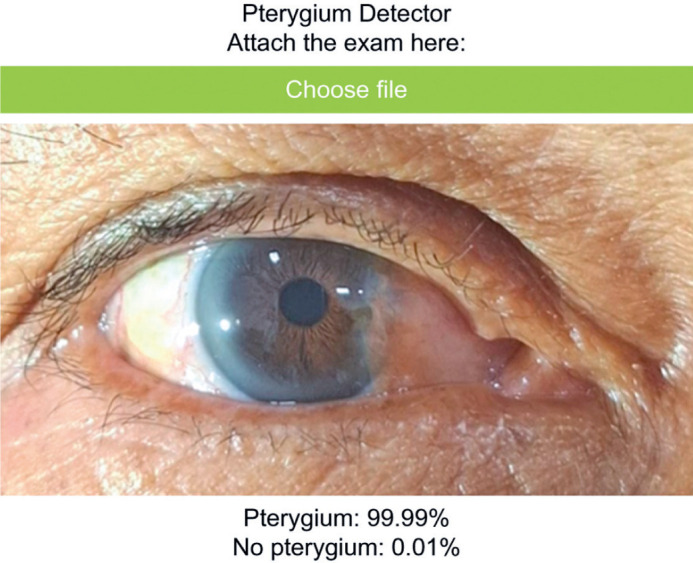
Interface and diagnostic output of the Gobvision AI platform.

Model development was conducted using the Gobvision AI platform, which is built on the MobileNet-V2 architecture and utilizes convolutional neural networks (CNNs) for efficient image classification. This web-based tool, accessible via https://www.gobvisionai.com/, enables clinicians to apply AI for diagnostic purposes without requiring advanced programming expertise. The platform was selected for its cost-effectiveness, accessibility, and the absence of alternative AI-based solutions specifically tailored for pterygium diagnosis (Figure 2).

The model was trained on anterior segment images using the platform’s default hyperparameters: 50 epochs, a batch size of 16, and a learning rate of 0.001. To improve generalizability and minimize overfitting, several data augmentation techniques were applied during training, including random rotations, horizontal and vertical flips, and adjustments in brightness and contrast. It is important to note that, as of this writing, the Gobvision AI model has not received formal regulatory approval, and its performance has not yet been validated in peer-reviewed studies.

### Statistical analysis

Model performance was evaluated using standard diagnostic metrics, including accuracy, sensitivity, spe cificity, F1 score, and the area under the receiver operating characteristic curve (AUC). Confidence intervals for these metrics were estimated using bootstrap resampling with 1,000 iterations.

The DL model outputs a probability score indicating the likelihood of pterygium. A case was classified as positive if the probability was ≥50% and negative if <50%. In addition, multiple logistic regression analysis was conducted to assess demographic variables associated with the occurrence of pterygium in the study population.

All statistical analyses were performed using DATAtab (2024), an online statistical tool developed by DATAtab e.U., Graz, Austria.

## RESULTS

A total of 62 patients (124 eyes) were initially enrolled in the study. However, 24 patients were excluded due to their nonparticipation in the second-stage anterior segment evaluation using slit-lamp examination. Thus, 38 patients (76 eyes) were included in the final analysis utilizing the DL model.

The mean age of the included participants was 45.61 yr (standard deviation=11.91). Among them, 57.89% were female, and 42.11% were male. Based on the ophthalmologist’s evaluation, the overall prevalence of pterygium was 46.05%. Sex-specific prevalence was 57.89% among females and 40.6% among males. Age-stratified analysis showed a pterygium prevalence of 46.87% among participants aged ≥50 yr and 45.44% among those aged <50 yr. A multiple logistic regression analysis revealed no statistically significant association between pterygium occurrence and sex (p=0.417) or age (p=0.884). Of the 38 patients diagnosed with pterygium, 12 (31.6%) presented with bilateral involvement.

**Figure 3:**
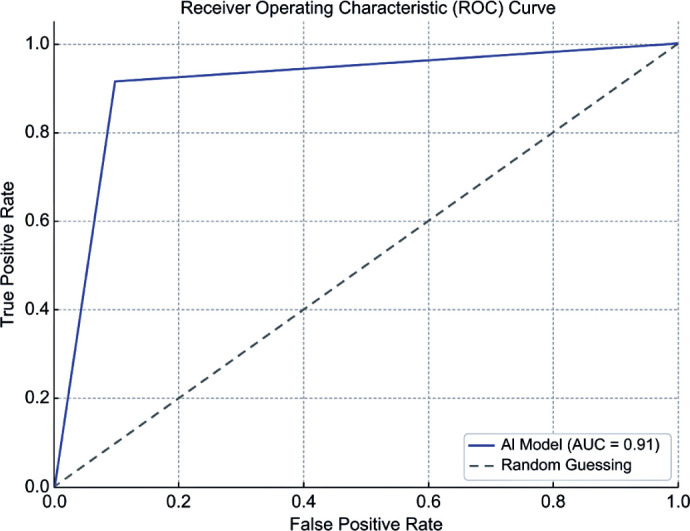
Receiver operating characteristic curve of the deep learning model.

**Figure 4:**
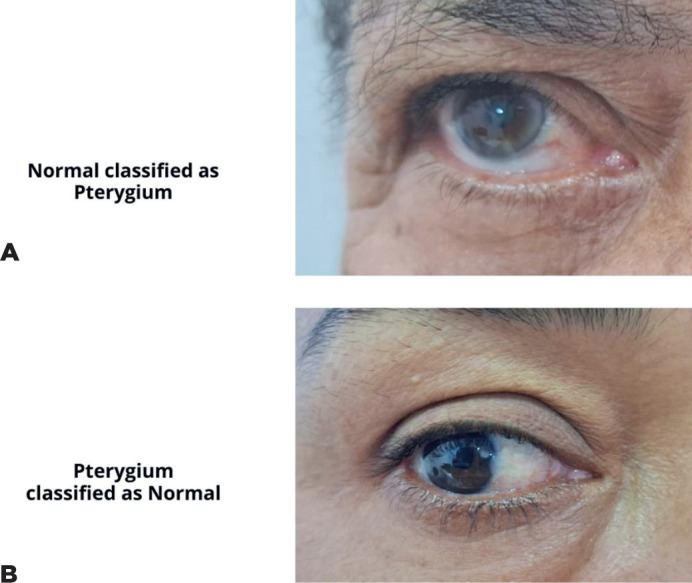
Examples of false-positive (A) and false-negative (B) cases from different patients.

**Figure 5:**
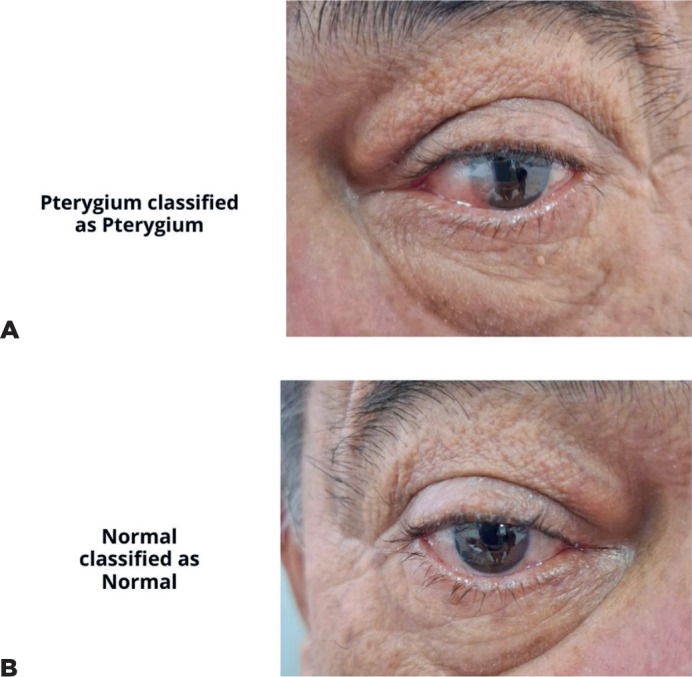
True-positive (A) and true-negative (B) eyes from the same patient.

The DL model achieved a sensitivity of 91.43% (95% confidence interval [95% CI], 76.94–98.20), specificity of 90.24% (95% CI, 76.87–97.28), positive predictive value of 88.46% (95% CI, 75.04–95.14), and NPV of 92.79% (95% CI, 81.28–97.45). The F1 score was 0.901, and the AUC was 0.91 (Table 1 and Figure 3). Representative examples of false-positive, false-negative, true-positive, and true-negative cases are illustrated (Figures 4 and 5).

**Table 1:** Predictive performance metrics of the AI model for pterygium detection

Metric	AI model
Sensitivity (%)	91.43 (76.94–98.20)
Specificity (%)	90.24 (76.87–97.28)
Positive predictive value (%)	88.46 (75.04–95.14)
Negative predictive value (%)	92.79 (81.28–97.45)
F1 score	0.901
Area under the curve	0.91

AI= artificial intelligence; AUC= area under the curve; NPV= negative predictive value; PPV= positive predictive value. Confidence intervals (CIs) for sensitivity, specificity, PPV, and NPV were estimated using bootstrap methods (1,000 replications). The AUC was calculated from the receiver operating characteristic curve. Multiple logistic regression analysis showed no significant association between sex (p=0.417) or age (p=0.884) and the occurrence of pterygium.

## DISCUSSION

Teleophthalmology has proven to be an effective support system for primary care in the diagnosis and management of ocular conditions in remote areas, particularly in regions such as the Amazon^(16)^. This approach facilitates ongoing monitoring of clinical outcomes without necessitating in-person consultations with ophthalmologists, which is especially valuable in settings with limited healthcare resources^(17)^.

AI has emerged as a promising tool for the diagnosis and management of pterygium^(11^,^12)^. Recent advancements in AI–particularly in machine learning and DL–have led to the development of automated systems for detecting and classifying pterygium, potentially enhancing diagnostic accuracy and efficiency^(14)^. Xu et al. implemented a DL-based intelligent diagnostic system using anterior segment photographs, achieving a high accuracy rate of 94.68% in classifying images into normal, observation, and surgical groups, along with excellent sensitivity, specificity, and F1-scores across categories^(15)^.

Similarly, Zheng et al.^(18)^ developed a lightweight AI model based on the MobileNet architecture, which demonstrated high sensitivity and specificity for pterygium diagnosis using anterior segment images. This model is particularly suitable for primary healthcare settings due to its efficiency and compatibility with mobile devices, supporting early screening and timely referrals. Gan et al.^(19)^ focused on detecting pterygium requiring surgical intervention using an ensemble DL model, while Zamani et al.^(20)^ proposed an enhanced CNN-based model, VggNet16-wbn, which achieved strong performance metrics, including 99.22% accuracy and 98.45% sensitivity, reinforcing the potential of DL approaches in developing effective screening tools.

Despite these advancements, several challenges hinder the widespread implementation of AI for pterygium diagnosis. These include variability in image quality, differences in clinical settings, and the need for large, diverse datasets to train robust AI models^(13^,^14)^. The heterogeneity of pterygium lesions–manifesting in variations in size, thickness, and vascularity–also complicates the development of standardized grading systems, limiting the consistency and accuracy of AI diagnoses^(19)^. Moreover, the computational demands of advanced AI systems pose a significant barrier, particularly in resource-constrained regions where such technologies are most urgently needed^(21)^. To address these challenges, this study proposes an accessible AI model that is freely available online and requires no programming expertise.

Despite the inherent limitations of this pilot study, the findings provide preliminary evidence that AI models can aid in identifying pterygium using smartphone-captured anterior segment images. The DL model achieved encouraging diagnostic performance in a limited dataset, with a sensitivity of 91.43%, specificity of 90.24%, and an area under the curve (AUC) of 0.91. These results are promising, especially considering the model’s ability to approximate the diagnostic accuracy of ophthalmologists^(22)^.

Although a fixed classification threshold of 50% was used, future studies could investigate the impact of adjusting this threshold to optimize sensitivity and specificity based on different clinical screening objectives.

One key limitation of this study is the relatively small sample size, which reflects the unique challenges of con ducting research in the Brazilian Amazon. Geographic and infrastructural barriers in this region restrict access and mobility, making participant recruitment difficult^(6)^. While this limitation may affect the generalizability of the findings and increase the risk of statistical bias, the study’s primary objective was to assess the feasibility and initial effectiveness of a DL model in a resource-limited and underserved setting^(23)^. The results suggest that this approach may be applicable in similar environments, particularly where access to specialists and advanced medical equipment is restricted.

During the early stages of the study, the research team encountered specific challenges, such as inconsistencies in initial image interpretations by health agents. These issues underscore the need for continuous training and routine evaluations to ensure diagnostic accuracy^(24)^. Another important limitation is the potential for selection bias, as patient inclusion was based on spontaneous visits to the BHU, which may not fully represent the broader population of the Amazon region^(25)^. This pilot study provides initial insights into the feasibility of using a DL model for pterygium diagnosis. Further research involving larger and more diverse populations is essential to validate these findings and assess model performance in varied clinical contexts^(25)^. This stepwise approach aligns with common trajectories in AI research, wherein early-stage studies assess feasibility under specific conditions before expanding to broader applications^(10)^.

Collaborations with local health authorities could be pivotal in adapting and validating these AI models for a wider range of ocular diseases^(26)^. Additionally, successful integration into the Amazon’s broader health care system will require embedding these tools within primary care workflows and providing ongoing support and training to healthcare professionals, including nonmedical health agents^(27)^. Such efforts may enhance healthcare delivery efficiency and reduce the burden on specialized services^(28)^.

Ultimately, these findings highlight the potential of collaborative efforts between health agents and ophthalmologists to leverage imaging and AI technologies in improving diagnostic capabilities in remote regions^(24)^. Continued investigation, including the surgical intervention phase and subsequent patient follow-up, will be essential to further validate the clinical utility of the proposed model^(25)^.

## Data Availability

The datasets generated and/or analyzed during the current study are available.

## References

[1] Chen J, Maqsood S, Kaye S, Tey A, Ahmad S (2014). Pterygium: Are we any closer to the cause?. Br J Ophthalmol..

[2] Shahraki T, Arabi A, Feizi S (2021). Pterygium: An update on pathophysiology, clinical features, and
management. Ther Adv Ophthalmol..

[3] Taylor R, Chen M, Jacobs DS, Jhanji V (2023). Update on evolving approaches for pterygia. EyeNet Mag..

[4] Chu WK, Choi HL, Bhat AK, Jhanji V (2020). Pterygium: new insights. Eye (Lond).

[5] Rezvan F, Khabazkhoob M, Hooshmand E, Yekta A, Saatchi M, Hashemi H (2018). Prevalence and risk factors of pterygium: a systematic review and
meta-analysis. Surv Ophthalmol..

[6] Fernandes AG, Salomão SR, Ferraz NN, Mitsuhiro MH, Furtado JM, Muñoz S (2020). Pterygium in adults from the Brazilian Amazon Region: prevalence,
visual status and refractive errors. Br J Ophthalmol..

[7] Zhong H, Cha X, Wei T, Lin X, Li X, Li J (2012). Prevalence of and risk factors for pterygium in rural adult
Chinese populations of the Bai nationality in Dali: The Yunnan Minority Eye
Study. Invest Ophthalmol Vis Sci..

[8] McCarty CA, Fu CL, Taylor HR (2000). Epidemiology of pterygium in Victoria, Australia. Br J Ophthalmol..

[9] Fotouhi A, Hashemi H, Khabazkhoob M, Mohammad K (2009). Prevalence and risk factors of pterygium and pinguecula: The
Tehran Eye Study. Eye(Lond).

[10] Food and Drug Administration (2023). Artificial intelligence and machine learning (AI/ML)-enabled medical
devices [Internet].

[11] Liu Y, Xu C, Wang S, Chen Y, Lin X, Guo S (2024). Accurate detection and grading of pterygium through smartphone by
a fusion training model. Br J Ophthalmol..

[12] Wan C, Shao Y, Wang C, Jing J, Yang W (2022). A novel system for measuring pterygium’s progress using deep
learning. Front Med (Lausanne).

[13] Gonçalves MB, Nakayama LF, Ferraz D, Faber H, Korot E, Malerbi FK (2023). Image quality assessment of retinal fundus photographs for
diabetic retinopathy in the machine learning era: a review. Eye (Lond).

[14] Chen B, Fang XW, Wu MN, Zhu SJ, Zheng B, Liu BQ (2023). Artificial intelligence assisted pterygium diagnosis: Current
status and perspectives. Int J Ophthalmol..

[15] Xu W, Jin L, Zhu PZ, He K, Yang WH, Wu MN (2021). Implementation and application of an intelligent pterygium
diagnosis system based on deep learning. Front Psychol..

[16] Torres E, Morales PH, Bittar OJ, Mansur NS, Salomão SR, Belfort RJ (2018). Teleophthalmology support for primary care diagnosis and
management. Med Hypothesis Discov Innov Ophthalmol..

[17] Larivoir NB, Camargo LM, Clemente BN, De Domenico RC, Camargo JS, Nascimento H (2022). Teleophthalmology postoperative evaluation of patients following
pterygium surgery in the Amazon. Pan Am J Ophthalmol..

[18] Zheng B, Liu Y, He K, Wu M, Jin L, Jiang Q (2021). Research on an intelligent lightweight-assisted pterygium
diagnosis model based on anterior segment images. Dis Markers.

[19] Gan F, Chen WY, Liu H, Zhong YL (2022). Application of artificial intelligence models for detecting the
pterygium that requires surgical treatment based on anterior segment
images. Front Neurosci..

[20] Zamani NS, Zaki WM, Huddin AB, Hussain A, Mutalib HA, Ali A (2020). Automated pterygium detection using deep neural
network. IEEE Access.

[21] Ciravegna G, Barbiero P, Giannini F, Gori M, Lió P, Maggini M (2023). Logic explained networks. Artif Intell..

[22] Li Z, Wang L, Wu X, Jiang J, Qiang W, Xie H (2023). Artificial intelligence in ophthalmology: the path to the
real-word clinic. Cell Rep Med..

[23] Ting DS, Cheung CY, Lim G, Tan GS, Quang ND, Gan A (2017). Development and validation of a deep learning system for diabetic
retinopathy and related eye diseases using retinal images from multiethnic
populations with diabetes. JAMA.

[24] Gulshan V, Peng L, Coram M, Stumpe MC, Wu D, Narayanaswamy A (2016). Development and validation of a deep learning algorithm for
detection of diabetic retinopathy in retinal fundus
photographs. JAMA.

[25] Guo J, Li B (2018). The application of medical artificial intelligence technology in
rural areas of developing countries. Health Equity.

[26] Burlina PM, Joshi N, Pacheco KD, Freund DE, Kong J, Bressler NM (2018). Use of deep learning for detailed severity characterization and
estimation of 5-year risk among patients with age-related macular
degeneration. JAMA Ophthalmol..

[27] Mursch-Edlmayr AS, Ng WS, Diniz-Filho A, Sousa DC, Arnold L, Schlenker MB (2020). Artificial intelligence algorithms to diagnose glaucoma and
detect glaucoma progression: translation to clinical
practice. Transl Vis Sci Technol..

[28] Abramoff MD, Lavin PT, Birch M, Shah N, Folk JC (2018). Pivotal trial of an autonomous AI-based diagnostic system for
detection of diabetic retinopathy in primary care offices. NPJ Digit Med..

